# A Chromosomal Inversion Unique to the Northern White-Cheeked Gibbon

**DOI:** 10.1371/journal.pone.0004999

**Published:** 2009-03-25

**Authors:** Lucia Carbone, Alan R. Mootnick, Tilo Nadler, Pierre Moisson, Oliver Ryder, Christian Roos, Pieter J. de Jong

**Affiliations:** 1 BACPAC Resources, Children's Hospital of Oakland Research Institute, Oakland, California, United States of America; 2 Gibbon Conservation Center (GCC), Santa Clarita, California, United States of America; 3 Endangered Primate Rescue Center, Cuc Phuong National Park, Ninh Binh Province, Vietnam; 4 Parc Zoologique et Botanique, Mulhouse, France; 5 Zoological Society of San Diego, San Diego, California, United States of America; 6 German Primate Center, Leibniz-Institut for Primate Research, Göttingen, Germany; Max Planck Institute for Evolutionary Anthropology, Germany

## Abstract

The gibbon family belongs to the superfamily Hominoidea and includes 15 species divided into four genera. Each genus possesses a distinct karyotype with chromosome numbers varying from 38 to 52. This diversity is the result of numerous chromosomal changes that have accumulated during the evolution of the gibbon lineage, a quite unique feature in comparison with other hominoids and most of the other primates. Some gibbon species and subspecies rank among the most endangered primates in the world. Breeding programs can be extremely challenging and hybridization plays an important role within the factors responsible for the decline of captive gibbons. With less than 500 individuals left in the wild, the northern white-cheeked gibbon (*Nomascus leucogenys leucogenys*, NLE) is the most endangered primate in a successful captive breeding program. We present here the analysis of an inversion that we show being specific for the northern white-cheeked gibbon and can be used as one of the criteria to distinguish this subspecies from other gibbon taxa. The availability of the sequence spanning for one of the breakpoints of the inversion allows detecting it by a simple PCR test also on low quality DNA. Our results demonstrate the important role of genomics in providing tools for conservation efforts.

## Introduction

Gibbons (family Hylobatidae) are small arboreal apes, which belong, together with humans and great apes, to the superfamily of Hominoidea. They inhabit tropical and semi-deciduous forests of Southeast Asia and small parts of South- and East-Asia [1]–[4]. Gibbons were the first to branch off from the other hominoids and display a set of characteristics distinctly different from great apes and humans. While gibbons are widely considered to form a monophyletic clade (Hylobatidae), there is no consensus about the taxonomy, phylogeny and evolutionary history within the family. Some of the earlier taxonomic manuscripts described the small apes to have two genera: *Symphalangus* (including one species) and *Hylobates* (including all other species) [4]. Subsequently, the family has been divided into four major clades, which were recognized as four subgenera [5] and eventually elevated to four genera [3], [6], [7]. This division takes into account the fact that species within each of the four major clades share a number of characteristics, most importantly a distinctive diploid chromosome number [2], [3], [8]: *Hoolock* (2n = 38), *Hylobates* (2n = 44), *Symphalangus* (2n = 50) and *Nomascus* (2n = 52). The genus *Hoolock* (hoolock gibbon) contains two species, while *Symphalangus* (siamang) consists of only a single species [3], [7]. The genera *Hylobates* (44 chromosome gibbons) and *Nomascus* (crested gibbons) comprise seven and five species, respectively [1], [9] (Table 1). The phylogenetic relationships among the four genera and among species have been examined at the level of morphology, taxonomy, behavior, vocalization, protein electrophoresis, molecular genetics and karyotyping, but this has not yet resulted in an unambiguous phylogeny [6], [10]. However, at least for crested gibbon taxa, a clear branching pattern following a north to south axis is depicted by mitochondrial sequence data [9].

**Table 1:** classification of gibbons as described in [3], [9], [29]

Genus	Species	Common name
*Nomascus*	*Nomascus concolor concolor*	Western black gibbon
	*Nomascus concolor lu*	Laotian black gibbon
	*Nomascus nasutus*	Eastern black gibbon
	*Nomascus hainanus*	Hainan gibbon
	*Nomascus leucogenys leucogenys*	Northern white-cheeked gibbon
	*Nomascus leucogenys siki*	Southern white-cheeked gibbon
	*Nomascus gabriellae*	Buff-cheeked gibbon
*Hoolock*	*Hoolock hoolock*	Western hoolock gibbon
	*Hoolock leuconedys*	Eastern hoolock gibbon
*Hylobates*	*Hylobates klossii*	Kloss' gibbon
	*Hylobates pileatus*	Pileated or capped gibbon
	*Hylobates moloch*	Javan, silvery, or moloch gibbon
	*Hylobates muelleri muelleri*	Eastern Müller's gibbon
	*Hylobates muelleri funereus*	Northern Müller's gibbon
	*Hylobates muelleri abbotti*	Abbott's gray gibbon
	*Hylobates agilis agilis*	Mountain agile gibbon
	*Hylobates agilis unko*	Lowland agile gibbon
	*Hylobates albibarbis*	Bornean white-bearded gibbon
	*Hylobates lar lar*	Malayan lar gibbon
	*Hylobates lar carpenteri*	Carpenter's lar gibbon
	*Hylobates lar entelloides*	Mainland lar gibbon
	*Hylobates lar vestitus*	Sumatran lar gibbon
*Symphalangus*	*Symphalangus syndactylus syndactylus*	Sumatran siamang
	*Symphalangus syndactylus continentis*	Malayan siamang

Some gibbon species are critically endangered and subject to captive breeding as part of the Species Survival Plan (SSP), with the ultimate goal of releasing physically and mentally healthy captive gibbons into a secure area of their native habitat. At this stage, to avoid undesirable hybridization with other taxa, it is essential that the release occurs in a proper location corresponding with the habitat of the same taxon [3]. Hybridization is detrimental to captive breeding or rehabilitation programs and it is also critical that hybridization does not occur when the animal is released into secure native habitat. On the other hand, the classification of gibbon species can be confusing and in some cases “visual” identification of a gibbon can be complicated by several factors (existence of different colors for the two sexes, variable color shades at different ages of the same gibbon, etc). The use of comparative genomics may therefore become important to assist with the classification of gibbons.

Comparisons between gibbon chromosomes and those of other primates soon revealed that gibbons present an extraordinary level of evolutionary chromosomal rearrangements, which obscured detection of most syntenic homologies with human and the great apes [8], [11]–[16]. This observation has drawn the interest of many scientists in the field of genome evolution. Nearly, all modes of chromosomal rearrangements observed in the karyotypic divergence of mammals have been recognized in gibbons (pericentric and paracentric inversions, chromosomal fission, Robertsonian and tandem fusion, reciprocal translocations). This feature is even more striking if one considers that the chromosomes of more distantly related primates, like Old World monkeys, share more similarities with humans than gibbons.

With the aim of identifying genetic bases of such instability we recently created a high-resolution map of synteny disruptions between the northern white-cheeked gibbon (*Nomascus leucogenys leucogenys*, NLE) and the human [14]. The northern white-cheeked gibbon is a critically endangered species (IUCN 2008, www.iucnredlist.org) with possibly less than 500 individuals left in the wild [T Nadler unpublished data], with successful captive breeding programs taking place. We report here the analysis of one particular inversion on NLE chromosome 7 described in the literature as “polymorphic” for individuals from the genus *Nomascus*. This assumption is based on scarce observations [17], [18] mostly because of difficulties in performing large scale cytogenetic studies on species from the genus since there are very low numbers of unrelated captive individuals [19], [20]. By taking advantage of the breakpoint sequence availability, we designed a test to discern presence or absence of this inversion simply by PCR. This approach does not require high quality DNA or chromosome preparations, and therefore additional individuals can be targeted, including those of which only low quality DNA is available. From our study samples we were able to show that only northern white-cheeked gibbons carry this inversion, with no evidence of it being polymorphic in this subspecies. As a result, our PCR test can become one of the tools for guiding some of the housing strategies in zoos and conservation centers with the advantage of not requiring cytogenetic experts.

## Results

### Cross-species analysis of breakpoint spanned by BAC CH271-263C9

**Figure 1:**
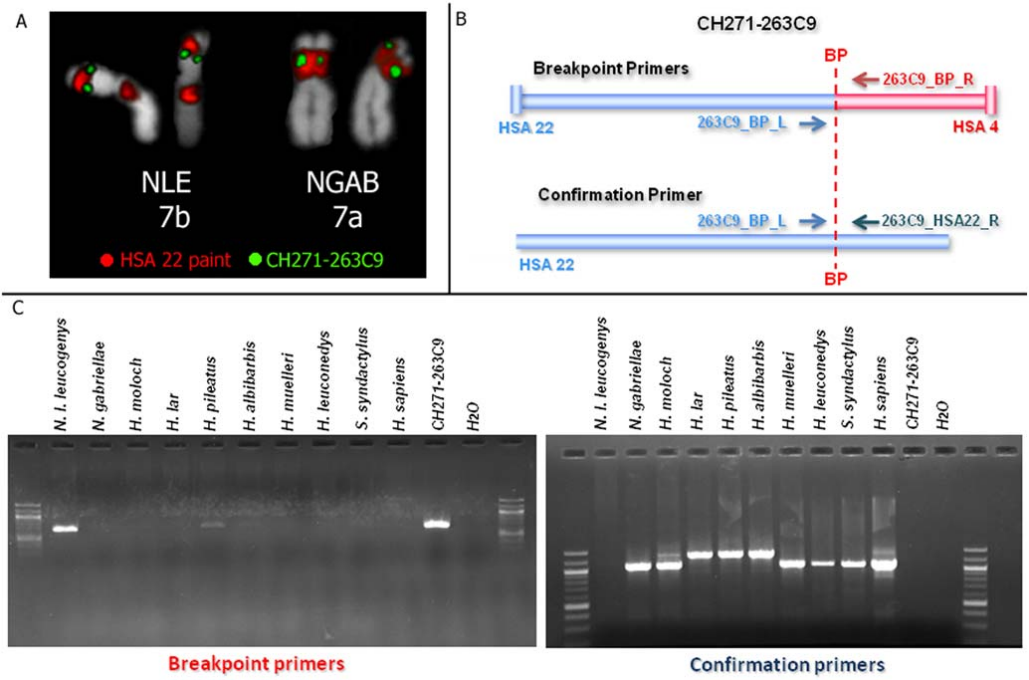
FISH and cross-species PCR with BAC clone CH271-263C9. A) Cohybridization of the whole chromosome paint of human chromosome 22 (red) and BAC CH271-263C9 (green) on NLE and NGAB *(N. gabriellae)* metaphases. These two species carry a different form of chromosome 7 (7b and 7a) due to an inversion which occurred in NLE; B) the image shows the cross-species PCR strategy; C) gel image summarizing the result of the cross-species PCR on different gibbon taxa using the primers illustrated in B. The weak band for *H. pileatus* is the result of a PCR artifact.

With the aim of looking at the mechanisms underlining the abundance of chromosomal rearrangements in gibbons, we recently established a physical map of synteny breakpoints between the northern white-cheeked gibbon and human. We identified 67 gibbon BAC (Bacterial Artificial Chromosome) clones spanning gibbon-human synteny breakpoints. We additionally fully sequenced a sample of these BACs to identify the position of the breakpoints at the base pair level. This sample included clone “CH271-263C9” (sequence accession CT954303) whose BES (BAC End Sequences) map onto human chromosomes 22 (HSA 22) and 4 (HSA 4), respectively. This BAC was identified by screening high density filters containing the NLE genomic BAC library (CHORI-271) using the mapping information obtained by array-painting [14]. Using FISH (fluorescence in situ hybridization), we verified that clone CH271-263C9 localizes on NLE chromosome 7b (NLE7b; Figure 1A) in correspondence of sequences homologous to human chromosome 22. We identified this junction to result from the inversion on NLE7 described by Couturier and Lernould [17]. In their study, chromosome banding was used to compare four crested gibbon taxa (*N. gabriellae* [buff-cheeked gibbon], *N. l. leucogenys*, *N. l. siki* [southern white-cheeked gibbon], *N. hainanus* [Hainan gibbon]) and to identify taxon-specific karyotypic traits. We decided to investigate the origin of this inversion in the gibbon lineage using a cross-species approach which takes advantage of the availability of the breakpoint site sequence. Our strategy is quite straightforward: two “breakpoint primers” (BP_primers) are designed on both sides of the breakpoint originally identified in NLE. When these primers are tested on the genomic DNA, they will generate an amplification product only if the breakpoint is present. An additional PCR experiment is required to rule out that the absence of an amplification product is due to a technical artifact: the breakpoint forward primer is combined with a “human specific” reverse primer (confirmation_primers), designed in the corresponding non-disrupted region of the human chromosome (Figure 1B). In the scenario where a breakpoint identified in NLE is not shared by another gibbon taxon, the PCR with the breakpoint primers should be negative as opposed to a positive result with the confirmation primers. Using this method, many samples can easily be tested per experiment, contingent on the availability of genomic DNA samples of minimal quality and quantity. In our first experiment, we used genomic DNA from nine individuals representing nine gibbon species in all four genera (Table 2, Figure 1C): *N. leucogenys*, *N. gabriellae*, *Symphalangus syndactylus* (siamang), *Hoolock leuconedys* (eastern hoolock gibbon), *Hylobates moloch* (Javan gibbon), *H. lar* (lar gibbon), *H. muelleri* (Mueller's gibbon), *H. pileatus* (pileated gibbon) and *H. albibarbis* (white-bearded gibbon).

**Table 2:** gibbon samples used in the study

Species	ID	material	origin/collector/storage	captive/wild born
*N. nasutus*	N12	skin	Tilo Nadler	wild born
*N. nasutus*	KimHy	skin	Tilo Nadler	wild born
*N. c. concolor*	NC1	skin	Lucy Tallents	wild born
*N. c. concolor*	NC2	skin	Lucy Tallents	wild born
*N. c. concolor*	NC3	skin	Lucy Tallents	wild born
*N. c. concolor*	GBP558	skin	Nicolas Lormee	wild born
*N. c. concolor*	GBP560	skin	Lucy Tallents	wild born
*N. c. concolor*	GBP561	skin	Lucy Tallents	wild born
*N. l. leucogenys*	GBP1005	blood	Duisburg Zoo	wild born, mother of 1007–1010
*N. l. leucogenys*	GBP1006	blood	Duisburg Zoo	wild born, father of 1007–1010
*N. l. leucogenys*	GBP1007	blood	Duisburg Zoo	offspring of 1005+1006
*N. l. leucogenys*	GBP1008	blood	Duisburg Zoo	offspring of 1005+1006
*N. l. leucogenys*	GBP1009	blood	Duisburg Zoo	offspring of 1005+1006
*N. l. leucogenys*	GBP1010	tissue	Duisburg Zoo	offspring of 1005+1006
*N. l. leucogenys*	GBP378	blood	Twycross Zoo	wild born
*N. l. leucogenys*	EPRC8-01	hairs	EPRC	wild born
*N. l. leucogenys*	EPRC8-02	hairs	EPRC	wild born
*N. l. leucogenys*	EPRC8-03	hairs	EPRC	wild born
*N. l. leucogenys*	EPRC8-04	hairs	EPRC	wild born
*N. l. leucogenys*	EPRC8-05	hairs	EPRC	wild born
*N. l. leucogenys*	EPRC8-08	hairs	EPRC	wild born
*N. l. leucogenys*	GBP1059	hairs	Mulhouse Zoo	wild born sire of 1059
*N. l. leucogenys*	GBP1060	hairs	Mulhouse Zoo	offspring of 1059+1061
*N. l. leucogenys*	GBP1061	hairs	Mulhouse Zoo	captive born
*N. l. leucogenys*	92	blood	Gladys Porter Zoo	wild born
*N. l. leucogenys*	101557	blood	Gladys Porter Zoo	captive born sibling to 101556
*N. l. leucogenys*	101556	blood	Columbus Zoo and Aquarium	captive born sibling to 101557
*N. l. leucogenys*	NLL605	blood	Gibbon Conservation Center	wild born
*N. l. leucogenys*	NLL607	blood	Gibbon Conservation Center	captive born
*N. l. leucogenys*	NLL97195	blood	Gibbon Conservation Center	offspring of NLL600+NLL601
*N. l. leucogenys*	NLL600	blood	Gibbon Conservation Center	captive born
*N. l. leucogenys*	NLL601	hairs	Gibbon Conservation Center	captive born
*N. l. siki*	EPRC9-03	tissue	EPRC	wild born
*N. l. siki*	EPRC9-05	hairs	EPRC	wild born
*N. l. siki*	EPRC9-06	hairs	EPRC	wild born
*N. l. siki*	GBP1062	hairs	Mulhouse Zoo	captive born
*N. l. siki*	GBP1063	hairs	Mulhouse Zoo	offspring of 1062
*N. gabriellae*	GBP410	blood	Zoologischer Garten Leipzig	wild born
*N. gabriellae*	EPRC10-01	hairs	EPRC	wild born
*N. gabriellae*	EPRC10-02	hairs	EPRC	wild born
*N. gabriellae*	EPRC10-04	tissue	EPRC	wild born
*N. gabriellae*	EPRC10-05	hairs	EPRC	wild born
*N. gabriellae*	EPRC10-06	hairs	EPRC	wild born
*N. gabriellae*	EPRC10-07	hairs	EPRC	wild born
*N. gabriellae*	EPRC10-08	hairs	EPRC	wild born
*N. gabriellae*	GBP1067	hairs	Mulhouse Zoo	wild born
*N. gabriellae*	195142	blood	Cincinnati Zoo and Botanical Gardens	captive born ½ related to 95141 & 94241. ¼ related to 96070
*N. gabriellae*	96075	blood	Los Angeles Zoo	captive born
*N. gabriellae*	96070	blood	Los Angeles Zoo	captive born
*N. gabriellae*	95141	DNA	Los Angeles Zoo	captive born sibling to 94241
*N. gabriellae*	94241	DNA	Los Angeles Zoo	captive born sibling to 95141
**Cross-genus PCR**				
**Species**	**ID**	**Material**	**origin/collector/storage**	**captive/wild born**
*S. syndactylus*	SS901	blood	Gibbon Conservation Center	captive born
*H. leuconedys*	HL307	blood	Gibbon Conservation Center	wild born
*H. moloch*	HMO894	blood	Gibbon Conservation Center	captive born
*H. agilis*	15353	blood	Henry Doorly Zoo	captive born
*H. lar*	9088	blood	Gladys Porter Zoo	captive born
*H. muelleri*	8136	blood	Gladys Porter Zoo	captive born
*H. albibarbis*	212067	blood	Louisiana Purchase Gardens Zoo	wild born
*H. pileatus*	HP120	blood	Gibbon Conservation Center	captive born

Abbreviation: EPRC; Endangered Primate Rescue Center; GBP; Gene Bank of Primates

The PCR results on this first set of samples revealed that the junction HSA 4-22 was present exclusively in NLE (Figure 1C). All the other species lacked breakpoint amplification whereas the region syntenic to human HSA 22 was amplified. We were not successful in amplifying the region homologous to HSA 4. Most significantly, the HSA 22-4 breakpoint was also absent in *N. gabriellae* indicating that this breakpoint may correspond with the cytogenetic inversion described by Couturier and Lernould [17].

**Figure 2:**
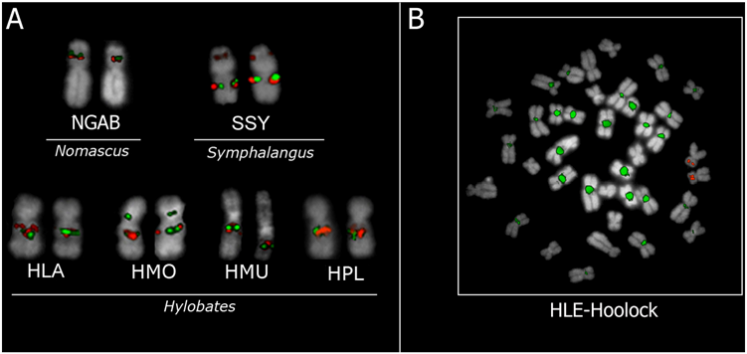
FISH on different gibbon species. A) FISH experiments on different gibbon species using as probe CH271-263C9 (red) and CH271-457L13 (green) which span the reciprocal breakpoints of the inversion on NLE7. B) BAC clone CH271-457L13 shows a peculiar pattern in hoolock with cross-hybridization on almost all the centromeres. Abbreviation used in the figure: NGAB is *N. gabriellae*, HLA is *H. lar*, HMO is *H. moloch*, HMU is *H. muelleri*, HPL is *H. pileatus*, HLE is *H. leuconedys*, SSY is *S. syndactylus*.

Our findings were further confirmed by FISH using two differentially-labeled BACs (CH271-263C9 & CH271-457L13) as probes. These gibbon clones map *in silico* to the same combination of syntenic regions on human chromosomes 22 and 4, as determined from the gibbon BES. The FISH results revealed that the two BACs map on different locations on the same chromosome for NLE, but co-localize for six other species representatives of three genera (Figure 2A). This is consistent with the two probes spanning the reciprocal breakpoints resulting from the NLE-specific inversion. Surprisingly clone CH271-457L13 generates an unusual pattern on the hoolock gibbon chromosomes, hybridizing to the centromeric regions of most chromosomes (Figure 2B). In humans and in the other gibbons this clone does not map on centromeric regions. As we were intrigued by this pattern we sequenced clone CH271-457L13 (at low coverage) using a shotgun approach and we looked for possible traces of satellites or repeats. The only satellite sequence we were able to identify was HSATI in the portion of the clone mapping on HSA 22. To understand if this satellite was responsible for the FISH pattern in *Hoolock*, we chose human BAC clones overlapping the same genomic region and performed further FISH analyses on hoolock gibbon chromosomes. None of the BACs produced the same pattern observed for clone CH271-457L13, instead a single spot was detected (data not shown). We therefore assumed that the gibbon clone contains some additional gibbon specific sequences (most likely repeats) that are present at high concentration at the centromere in the hoolock gibbon. This is additional evidence that regions overlapping with synteny breakpoints are often rich in repeats and present some kind of plasticity whose outcome can vary in different species [14].

### Junction HSA 22-4 is specific for NLE

Our preliminary investigation showed that this inversion is specific for NLE in our small set of samples. We then needed to exclude the possibility that this inversion is polymorphic in NLE and/or closely related taxa. We did not find any study including an adequate number of individuals to reach a conclusion. Nevertheless this rearrangement has been defined polymorphic [18]. We, therefore, tested the “BP_primers” and the “confirmation_primers” on 51 crested gibbon DNA samples obtained from blood, tissue or hair (Table 2, materials and methods). The results of this extensive investigation confirmed that the breakpoint HSA22-4 is exclusive for the northern white-cheeked gibbon. Our sample also includes five southern white-cheeked gibbons (*N. l. siki*, NLS), three of which were “wild-born” (Table 2). Couturier and Lernould [17] reported in their study that northern and southern white-cheeked gibbons share the same form of chromosome 7. The PCR test with the breakpoint primers did not generate an amplification product from the NLS samples, while amplification with the confirmation primers was obtained. This result indicates that the five NLS do not carry the inversion found in NLE.

### Multi-species sequence analysis of the HSA 4-22 breakpoint site

DNA sequences of the amplification product of the undisrupted region in different gibbon species (*H. agilis* (agile gibbon), *H. moloch*, *S. syndactylus* and *H. leuconedys*) gave us the opportunity to reconstruct and analyze the inferred ancestral chromosomal site, which in NLE was then disrupted by the inversion. This analysis demonstrated that the ancestral locus contains two Alu sequences, which are also present in human. The inversion breakpoint disrupted one of the Alu elements that recombined with a LTR (Long Terminal Repeat) element located on the segment homologous to human chromosome 4 (Figure S1). From the study of the other synteny breakpoints of the northern white-cheeked genome, we know that Alu elements are the most represented repeats at the breakage sites (Carbone unpublished data). This is not surprising, as it is well known that *Alu-Alu recombination* events are often responsible for chromosomal rearrangements in primate evolution and human genomic disorders [21], [22]. Interestingly, one example of a gene deletion due to an *Alu-Alu recombination* event has been recently characterized in detail in gibbons [23].

Following inspection of the human reference assembly revealed that this breakpoint most likely did not result in a gene disruption on chromosome 22 or chromosome 4 in the northern white-cheeked gibbon.

## Discussion

In this study we present the analysis of a chromosomal inversion that differentiates the northern white-cheeked gibbon (NLE) from the other gibbon taxa. This chromosomal rearrangement has previously been defined as “polymorphic” for the northern white-cheeked gibbon [17], [18] without any concrete population analysis done [24]. Our investigation is the first study with a substantial number of *Nomascus* individuals, taking into consideration that these are very rare apes. All tested NLE individuals were homozygous for this inversion. We additionally found that southern white-cheeked gibbons (NLS) do not share this inversion with NLE, in disagreement with Couturier and Lernould [17]. NLS has a different geographic distribution than NLE, as they inhabit Southern Laos, central Vietnam and possibly northeast Cambodia [25] whereas NLE is found in Northern Laos, Northwestern Vietnam and Southeastern China [3]. Following our results, it seems likely that NLS does not carry the NLE7 inversion. To explain this difference with the finding of Couturier and Lernould we hypothesize a sampling error. There are many reasons why we were intrigued by this chromosomal rearrangement and pursuit its characterization. First, this inversion is not shared by other members of the same genus and it is more likely that the most recent chromosomal rearrangement occurred in NLE. Taking into consideration the chromosomal theory of speciation, we wondered if this inversion could have been responsible for causing reproductive isolation in this gibbon population and then drive a speciation event [26]. Further studies on sequence divergence within the population will be necessary to exploit this possibility. Second, an important outcome of our study is that, given its taxon-specificity, this chromosomal breakpoint may be use as one of the markers to distinguish the northern white-cheeked gibbon from other taxa or hybrids, serving as a tool for conservation purposes. Several cooperative breeding programs are currently in place for endangered gibbon species. Breeding gibbons in captivity can have the ultimate goal of releasing gibbons in a protected native habitat after creating a viable gene pool [3]. Many breeding and conservation centers have difficulties in identifying which gibbon species or subspecies that they house, and in determining which individual gibbon would benefit in their breeding program. This is a very important issue for species as rare as the northern white-cheeked gibbon. In a recent publication, Mootnick [3] commented on all the complications that can arise when conservation or rescue centers need to identify some of the gibbon taxa. One of the confusing factors existing in gibbon taxonomy was ambiguous or incomplete descriptions found in some publications. Karyotyping represents a powerful method to distinguish between different species where other morphological traits are unclear. Unfortunately, most breeding centers do not have the training or equipment for genetic analysis. Another limitation is chromosomal preparation and the availability of fresh blood from each individual. In the case of endangered species, this becomes a recurring limiting factor to take into account. Recently a new approach called “DNA barcoding” [27], [28] emerged in the conservation and forensic community, demonstrating the need to complement classic taxonomy with other tools. This method proposes the use of a single locus that can be amplified through specific primers and sequences; sequences are then introduced in a database in order to classify species or identify new species. Since it was introduced, this approach generated controversial opinions in the scientific community and many experts argued that DNA barcoding could produce misleading results if not accompanied by a full taxonomic revision [26]. The sequence that is used to “barcode” animals is the mitochondrial *cytochrome oxidase I (coxI)* gene which seems to have the features required for such analysis: it contains sufficient variability between species, it is short enough to be analyzed in one experiment and it contains conserved regions that can be used to design universal primers [27]. The clear advantage of this method is the need for minimal technical support, as only one PCR reaction is necessary. However, by analyzing only a maternally inherited gene, possible hybrids remain undetected.

Our approach can be seen as a combination between classic cytogenetics and the DNA barcoding method. In our case, only few PCR experiments are required to discern similar species but the target of such amplification is a synteny breakpoint and not a gene. One of our long term goals is to identify at least one species-specific marker for each gibbon species in order to make them available to the conservation community. It is noteworthy that most of our PCR experiments were done on “low quality” DNA samples but were still successful. This feature is quite important when only materials collected in the wild are available, which are mostly incompatible with cytogenetic analysis. The use of a chromosomal rearrangement as a marker has the advantage of being uncoupled from population sequence variations but based on a simple presence/absence analysis. Additionally, as we are planning to characterize the sequences spanning the complete set of chromosomal rearrangements for all the gibbon species, these data will help to clarify the phylogenetic relationships between gibbon species. We are aware, however, that chromosomal rearrangements cannot be isolated criteria to identify different gibbon taxa and the complete assessment has to be based also on morphological and other molecular data. Nevertheless, our PCR approach represents a first screening step to retrieve information that otherwise would require fresh blood samples and cytogenetic expertise, which in many cases are not available.

## Materials and Methods

### Fluorescent in situ hybridization

Chromosome preparations for all the gibbon species in this study were obtained from peripheral blood following standard procedures. Briefly, blood was incubated with cell culture media and phytohemagglutinin (GIBCO) for 72 hours (37°C, 5 % CO_2_). Colcemid was then added (final concentration 0.05 ug/ml) and cells were harvested after a 1 hour incubation. Cells were spun down by centrifugation, the media was discarded and the pellet was resuspended in 8ml of hypotonic solution. After incubating for 20 minutes, the standard fixative solution (1 part Acetic Acid, 3 parts Methanol) was added and cells were centrifuged at 2500rpm for 5 minutes. The pellet was washed with fixative solution and cells were kept at 4°C overnight.

DNA from BACs was extracted using PureLink Miniprep kit (Invitrogen) as previously described [14]. Images were acquired using Nikon 80i microscope, equipped with CCD camera Cool Snap HQ^2^ (Photometrics) and software Nis Elements Br (NIKON). Elaboration of the images was done using Photoshop.

### Samples

For the present study, DNA from each individual of *S. syndactylus*, *H. leuconedys*, *H. moloch*, *H. agilis*, *H. lar*, *H. muelleri*, *H. albibarbis* and *H. pileatus* as well as from 2 individuals of *N. nasutus* (eastern black gibbon), 6 individuals of *N. concolor* (western black gibbon), 24 individuals of *N. l. leucogenys*, 5 individuals of *N. l. siki* and 14 individuals of *N. gabriellae* were examined (Table 2). All study specimens were identified by pelage coloration, additional external characteristics, and geographical distribution for gibbons living in their native habitat. Moreover, mainly wild-born gibbons or samples collected during field surveys in local houses were tested. Captive-born specimens were only studied if the species identification of their parents was possible. Total genomic DNA from blood and tissue was extracted using the DNeasy Blood & Tissue Kit from Qiagen. Hair follicle cells were directly implemented into the PCR reactions after they were washed with sterile water and 90% ethanol. Blood and tissues were obtained in agreement with protocols reviewed and approved by the Institutional Animal Care and Use Committee.

### Cross-species PCR

To easily identify the breakpoint in various gibbon taxa, a PCR system with two different PCR reactions were established. To verify the breakpoint, primers 263C9_BP_L (5′-ATTCGTAAGGCAGTGAGATG-3′) and 263C9_BP_R (5′-GGTTTGTCCTCACTGGAATA-3′) were used, whereas to confirm its absence, primer 263C9_BP_L was combined with primer 263C9_HSA22_R (5′-CTGAGAACTGTATGGAAGACTG-3′) (Figure 1B). For both reactions, identical PCR conditions including a pre-denaturation step at 94°C for 2 min., 30 cycles each with 94°C for 30 sec., 55°C for 30 sec. and 72°C for 1 min., and a final extension step at 72°C for 10 min. were applied. Results of PCR amplifications were checked on 1% agarose gels.

## Supporting Information

Figure S1 Alignment showing the region orthologous to the BOSR in gibbon individuals representative of each of the four genera (NLE, HAGI, HMO, HLE, SSY). The figure displays the region homologous to human chromosome 22. The ancestral arrangement comprises two Alu elements (light blue and green) from the subfamily S, the breakpoint (BP) and a LINE element (violet). The LTR element (orange) was inserted only in NLE after the chromosomal rearrangement occurred. Primer sequences are indicated in bold.
(0.11 MB DOC)
