# Supplementary figures and images for: A Chromosomal Inversion Unique to the Northern White-Cheeked Gibbon
Source: PLoS One. 2009 Mar 25;4(3):e4999. doi: 10.1371/journal.pone.0004999 (PMC2656618; doi:10.1371/journal.pone.0004999)

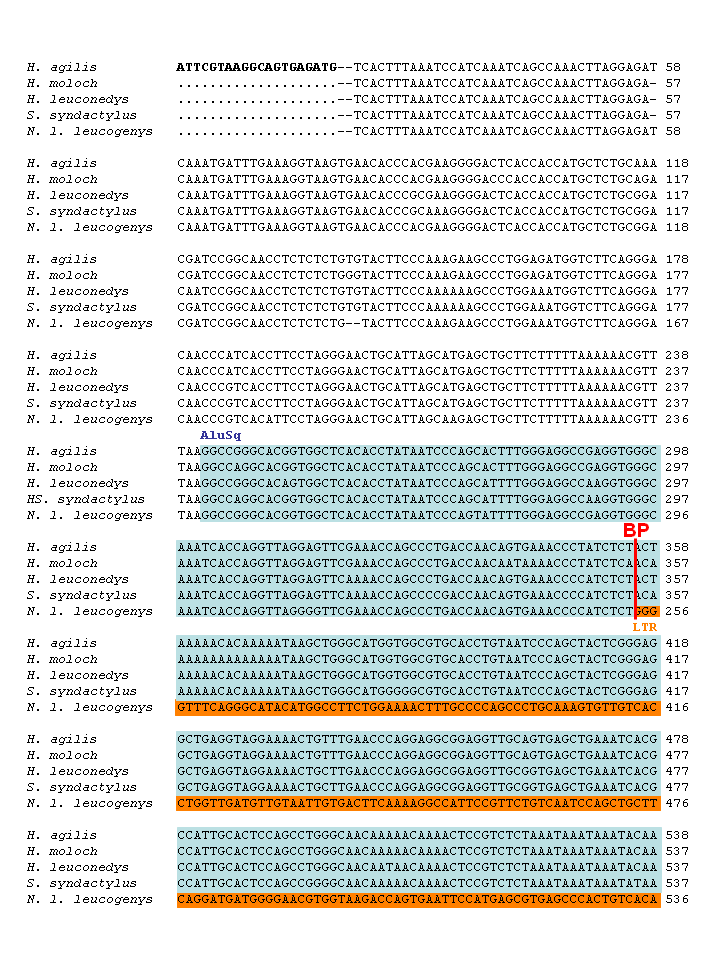


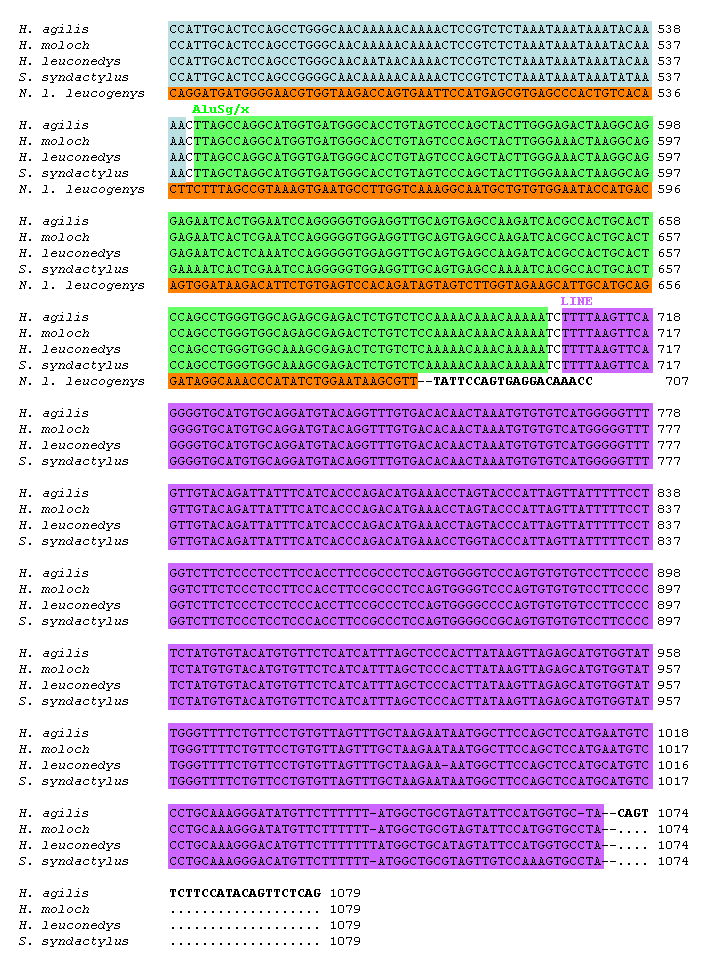

Supplement: Figure S1 — Alignment showing the region orthologous to the BOSR in gibbon individuals representative of each of the four genera (NLE, HAGI, HMO, HLE, SSY). The figure displays the region homologous to human chromosome 22. The ancestral arrangement comprises two Alu elements (light blue and green) from the subfamily S, the breakpoint (BP) and a LINE element (violet). The LTR element (orange) was inserted only in NLE after the chromosomal rearrangement occurred. Primer sequences are indicated in bold. (0.11 MB DOC) [file pone.0004999.s001.doc]
